# Hyperspectral Detection of a Subsurface CO_2_ Leak in the Presence of Water Stressed Vegetation

**DOI:** 10.1371/journal.pone.0108299

**Published:** 2014-10-20

**Authors:** Gabriel J. Bellante, Scott L. Powell, Rick L. Lawrence, Kevin S. Repasky, Tracy Dougher

**Affiliations:** 1 Department of Land Resources and Environmental Sciences, Montana State University, Bozeman, Montana, United States of America; 2 Department of Electrical and Computer Engineering, Montana State University, Bozeman, Montana, United States of America; 3 Department of Plant Sciences and Plant Pathology, Montana State University, Bozeman, Montana, United States of America; Xiamen University, China

## Abstract

Remote sensing of vegetation stress has been posed as a possible large area monitoring tool for surface CO_2_ leakage from geologic carbon sequestration (GCS) sites since vegetation is adversely affected by elevated CO_2_ levels in soil. However, the extent to which remote sensing could be used for CO_2_ leak detection depends on the spectral separability of the plant stress signal caused by various factors, including elevated soil CO_2_ and water stress. This distinction is crucial to determining the seasonality and appropriateness of remote GCS site monitoring. A greenhouse experiment tested the degree to which plants stressed by elevated soil CO_2_ could be distinguished from plants that were water stressed. A randomized block design assigned Alfalfa plants (Medicago sativa) to one of four possible treatment groups: 1) a CO_2_ injection group; 2) a water stress group; 3) an interaction group that was subjected to both water stress and CO_2_ injection; or 4) a group that received adequate water and no CO_2_ injection. Single date classification trees were developed to identify individual spectral bands that were significant in distinguishing between CO_2_ and water stress agents, in addition to a random forest classifier that was used to further understand and validate predictive accuracies. Overall peak classification accuracy was 90% (Kappa of 0.87) for the classification tree analysis and 83% (Kappa of 0.77) for the random forest classifier, demonstrating that vegetation stressed from an underground CO_2_ leak could be accurately discerned from healthy vegetation and areas of co-occurring water stressed vegetation at certain times. Plants appear to hit a stress threshold, however, that would render detection of a CO_2_ leak unlikely during severe drought conditions. Our findings suggest that early detection of a CO_2_ leak with an aerial or ground-based hyperspectral imaging system is possible and could be an important GCS monitoring tool.

## Introduction

### Geologic Carbon Sequestration

Post-industrial atmospheric carbon dioxide (CO_2_) concentration has risen from 280 ppm to over 380 ppm [1]–[6]. Atmospheric CO_2_ absorbs and reemits radiation energy from the Earth’s surface causing a warming of the surface environment [7]. Global warming could drastically reshape the Earth’s climate and environment [8]–[12]. Geologic carbon sequestration (GCS) is a potential climate change mitigation strategy that captures point source CO_2_ emissions from industrial sources and stores them in large sub-surface reservoirs. GCS, conceptually, has the potential to store gigatonnes (Gt) of CO_2_, which would otherwise be released into the atmosphere, into specialized geologic formations for long-term storage [13]–[19].

There is a mandate to monitor GCS sites for CO_2_ leakage to ensure the efficacy of this technology, given that it is on the brink of commercial-scale deployment [14], [19], [20]–[22]. Remote sensing is being investigated as a possible cost-effective, large-area monitoring method to detect surface CO_2_ leaks at GCS sites [23]–[24]. A leak from a GCS site would not only compromise the viability of this technology as a climate mitigation strategy, but it could also threaten the safety of the surrounding environment and inhabitants at the surface. The 1986 eruption of CO_2_ at Lake Nyos, Western Camaroon, killed over 1,700 people and demonstrated that a large pneumatic CO_2_ explosion can have devastating consequences [25]. Natural sources of CO_2_ leakage from the Long Valley Caldera in California have caused extensive forest mortality [26]–[28].

Elevated CO_2_ levels in soil are known to cause anoxic conditions in plant roots [26], [29], thereby interfering with plant respiration and inducing a stress response that could possibly be remotely sensed using aerial imagery [24]. Vegetated areas at GCS sites, presumably, could act as bellwethers to signal operational inefficiencies in hazardous CO_2_ leak scenarios. Vegetation status and seasonality will determine when the remote detection of vegetation stress caused by elevated soil CO_2_ would be feasible. Soil water availability is highly spatially variable, and water stressed vegetation could appear spectrally similar to vegetation stressed by elevated soil CO_2_. Drought is another common environmental occurrence that can have lasting impacts on whole land regions and can be short-lived or persist for years. Regardless of duration and spatial extent, monitoring GCS sites with remote sensing data would require discrimination between water and CO_2_ stress to accurately identify CO_2_ leaks.

### Remote Sensing as a Monitoring Technique for GCS Sites

A variety of monitoring techniques are being investigated for efficiency and accuracy at detecting a CO_2_ leak from a subsurface storage reservoir [30]. Most methods tend to be expensive and resource intensive, while providing only limited spatial coverage. Remote sensing, alternatively, has the potential to provide near instantaneous monitoring over large swaths of land. Aerial imaging over GCS sites would be relatively inexpensive and might be performed with limited labor requirements. Regions of elevated soil CO_2,_ if accurately detected with remote sensing equipment, could be further investigated with instruments on the ground to properly diagnose the leak source. Early leak detection would allow site managers to take immediate remediation measures to prevent further CO_2_ leakage, while minimizing safety risk and economic loss. GCS monitoring could tolerate false positives, within reason, whereas, a false negative resulting in a missed CO_2_ leak could have serious consequences.

### Hyperspectral Remote Sensing for Plant Stress Detection

Hyperspectral sensors collect nearly continuous spectral data across narrow channel widths throughout the visible and infrared portion of the electromagnetic (EM) spectrum (400–2500 nm). Spectral signatures or spectra can be used to detect subtle patterns in vegetation reflectance to better understand the physiological condition of plants. Plant pigment concentrations, leaf cellular structure, and leaf moisture content can be discerned with hyperspectral data to assess the overall health of vegetation and characterize plant stress [31]–[32].

Hyperspectral remote sensing has been used to detect and characterize numerous types of vegetation stressors within the visible and infrared portion of the EM spectrum. Hyperspectral data have been analyzed to model plant stress caused by elevated soil CO_2_ [33], water stress [34]–[36], insect and pest invasion [37]–[40], heavy metal contamination [22], salinity stress [41], nutrient levels and crop status [42]–[43]; ozone exposure [44]; and natural gas leaks [45]. Certain spectral regions are known to be especially sensitive, because they relate to the specific chemical and physical responses of plants to stress.

Low reflectance in the visible portion of the EM spectrum (400–700 nm) is determined primarily by chlorophyll *a* and *b* pigment abundance and absorption, while high reflectance in the NIR (750–2500 nm) is governed by the structural, spongy mesophyll contained in plant leaves [31], [46]–[49]. Chlorophyll absorption is highest in the visible blue (400–500 nm) and middle red (near 680 nm), therefore these regions are not sensitive to subtle changes in chlorophyll content, because the spectral signal is saturated [46], [50]. Absorption by chlorophyll pigments is weakest in the visible green – orange (500–620 nm) and the far visible red (700 nm), therefore, these wavelengths are sensitive to stress and subtle changes in chlorophyll content [46], [51]. Red edge indices are frequently used to detect stress at the boundary of the far red and NIR to detect changes in chlorophyll content. The red edge is a reflectance spike caused by steep transition from low reflectance in the far red to high reflectance in the NIR, which is used as reference because it is determined by the physical structure of plant leaves. Far red reflectance (near 700 nm) is known to increase as a plant exhibits a physiological stress response of reducing chlorophyll content to down regulate photosynthesis, thereby reflecting proportionally more potentially damaging photosynthetically active radiation compared to healthy vegetation, which can utilize all of that incoming energy. Vegetation stress caused by depleted oxygen concentrations around the roots from natural gas leaks and elevated soil CO_2_ has been detected in the red edge region of the EM spectrum [23]–[24], [33], [45], [52]. Water stress has also been found to cause reflectance changes related to chlorophyll pigment concentrations in the red edge [53]–[54].

Shifts of the red edge 5–10 nm towards shorter wavelengths, termed the “blue shift”, has also been associated with a decline in chlorophyll absorption for stressed vegetation [32]–[33], [45], [52], [55]–[58]. This phenomenon has been associated with the early detection of vegetation stress by hyperspectral remote sensing instruments. The sensitivity of hyperspectral instruments to detect vegetation stress early, and perhaps even before it is visible to the human eye, has distinct advantages over conventional monitoring methods, because land managers could then take quicker ameliorative action to minimize losses to valuable resources [37].

Vegetation reflectance in the green portion of the EM spectrum is associated with xanthophyll pigments in addition to chlorophyll. Xanthophyll pigments contained within plants’ palisade mesophyll perform photoprotective roles that also influence photosynthetic radiation use efficiency and are sensitive to stress [42], [59]. Vegetation reflectance within the green, therefore, will increase in response to stress to down regulate photosynthetic activity because of both xanthophyll pigment activity and decreases in chlorophyll content. The Photochemical Reflectance Index (PRI) is a narrow-band, hyperspectral index ratioing green reflectance and is known to be sensitive to xanthophyll pigment activity. The PRI has been used as an early indicator of water stress in plants caused by drought conditions [34]–[36], [43].

Water stress responses in vegetation have also been detected in spectral water absorption features in the shortwave infrared (SWIR) portion of the EM spectrum. Reflectance within the 1300–2500 nm wavelengths has been associated with leaf water content and has been observed to increase as a primary response of plants to water stress [53]. Visible spectrum reflectance has also been shown to increase, but only as a secondary plant response to water stress, in which chlorophyll pigment concentration decreases as a mechanism of down regulating photosynthesis during prolonged dehydration in vegetation [53]–[54]. Spectral measurements of leaf water content in the SWIR, therefore, directly monitors for plant dehydration and would be a more immediate method of detecting a plant’s response to water stress. The primary response of plants to drought within the water absorption bands also could possibly be spectrally distinct from other types of physiological stressors, including stress caused by elevated soil CO_2_.

Our greenhouse experiment objectives were (1) to evaluate if elevated soil CO_2_ stress can be detected in alfalfa plants using hyperspectral data, and if so how quickly; (2) to determine if CO_2_ stress is spectrally distinguishable from water stress since water availability is likely to be spatially variable at GCS sites and water stressed vegetation will likely co-occur with CO_2_ stressed vegetation during a CO_2_ leak scenario; and (3) to determine if CO_2_ stress is distinguishable during drought conditions when all vegetation is water stressed during a CO_2_ leak scenario. It is still unknown whether the plant stress signal caused by elevated soil CO_2_ is spectrally distinguishable from other forms of plant physiological stress. Water stress or drought conditions in particular, are common natural occurrences that might confound a CO_2_ stress signal if both plant physiological stressors coincide. Remote CO_2_ leak detection might therefore be problematic in the context of GCS monitoring. Although this experiment does not directly test the use of hyperspectral imaging for CO_2_ leak detection in a real-world aerial monitoring context, understanding the spectral discernability between stress caused by elevated soil CO_2_, water stress, and their interaction is important for determining the appropriate times and conditions in which remote sensing image acquisition should commence over GCS sites.

## Methods

### Data Collection

**Figure 1:**
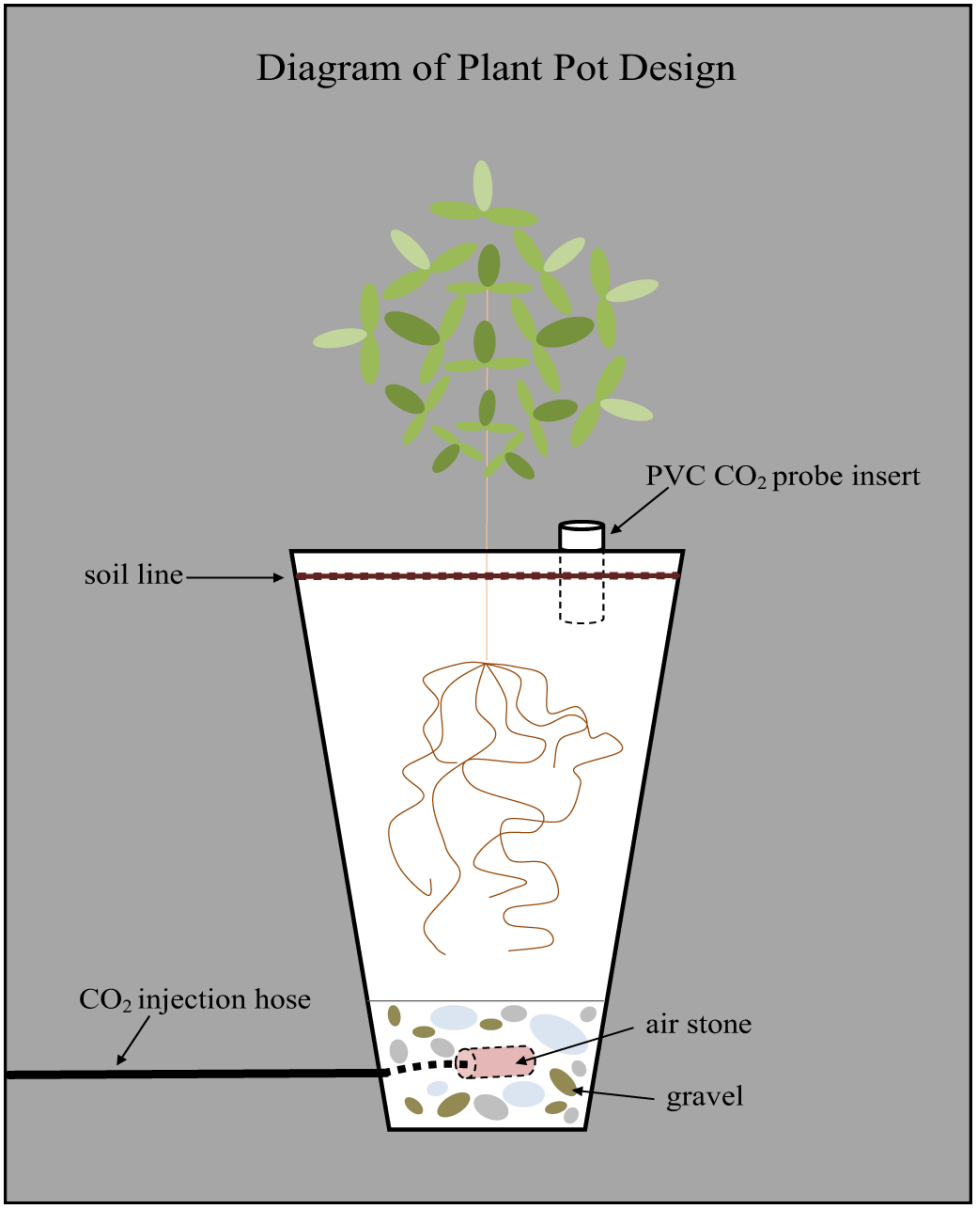
Diagram of a plant pot for the greenhouse experiment.

Alfalfa (*Medicago sativa*) seeds were planted on June 1, 2010, to obtain 40 mature plants for this greenhouse experiment. Each alfalfa seedling was transplanted into a 45 cm tall, 9.6-l tree pot (Stuewe & Sons, Inc.). These tree pots were modified in several ways prior to planting in order to facilitate CO_2_ injection into each individual plant pot and to best emulate a CO_2_ leak scenario from a GCS site. The drainage holes at the bottom of each pot were sealed using plastic wrap, silicone, and Gorilla tape to minimize injected CO_2_ from readily escaping through the bottom of the pots. The pots were then filled with 1.0 l of pea-sized gravel placed on top of a layer of gauze cotton cloth that covered drain holes at the bottom of each pot. A 0.6-cm diameter tube was inserted into each plant pot above the bottom 5-cm layer of gravel. The tube terminated in a 5-cm long, ceramic air stone (Top Fin) intended to disperse the CO_2_ gas radially, as opposed to being released from a single point source. The embedded air stone plumbing was topped with an additional l of gravel to assist in the dispersion of the CO_2_ gas throughout each pot and to allow for adequate water drainage from the soil (Figure 1).

Soil was placed on top of another layer of gauze above the gravel to keep the media separate. The soil used in the pots was a mixture of equal parts loam, washed concrete sand, and Canadian Sphagnum peat moss. Additionally, AquaGro 2000 wetting agent was blended into the soil at a ratio of 0.5 kg/m^3^ soil mix. The soil mix was pasteurized with aerated steam at 70°C for 60 minutes. A 10 cm long piece of 2.54 cm diameter PVC pipe was inserted 5 cm down into the surface of the soil of every plant to allow for immediate access for soil CO_2_ concentration measurements with a Vaisala GMP221 probe to verify injection. The alfalfa plants were watered with approximately 300 ml of water at 0800 hours every Monday, Wednesday, and Friday. They were fertilized twice a week with a ^1 gm^/_0.5 l_ dilution of NPK fertilizer (20-10-20) every Wednesday and Friday until CO_2_ injection began. The greenhouse temperature averaged 21.25±1.60°C over the course of the experiment. Sixteen hours a day of supplemental artificial lighting was provided using 1000 watt metal halide growth bulbs (GE Multi-Vapor MVR 1000/C/U) from 0500 to 2100 hours. Greenhouse integral photosynthetic photon flux measured within each of the treatment blocks ranged from 9.41 to 15.79 mol/m^2^/day on peak sunny days and 0.09 to 0.11 mol/m^2^/day on cloudy days (Apogee Instruments Line quantum sensors).

Five treatment blocks were developed, containing eight plants each (two replicates of each of four treatment groups per block). The four treatment groups included: (1) CO_2_ injection (I); (2) water stress (WS); (3) an interaction group that was subjected to both water stress and CO_2_ injection (WSI); and (4) adequate water and no CO_2_ injection (C). Each treatment block contained two plants from each treatment group. CO_2_ gas was delivered to the plant pots through a plumbing manifold that used equal lengths of tubing between each of the injected plants and a 2-stage pressure reducing regulator (Smith) at the CO_2_ tank, which maintained constant pressure and did not require adjustment as the pressure within the CO_2_ storage cylinder decreased over time. The gas was delivered from cylinders containing 50 lbs. liquid CO_2_ at a constant pressure of 15 psig.

**Figure 2:**
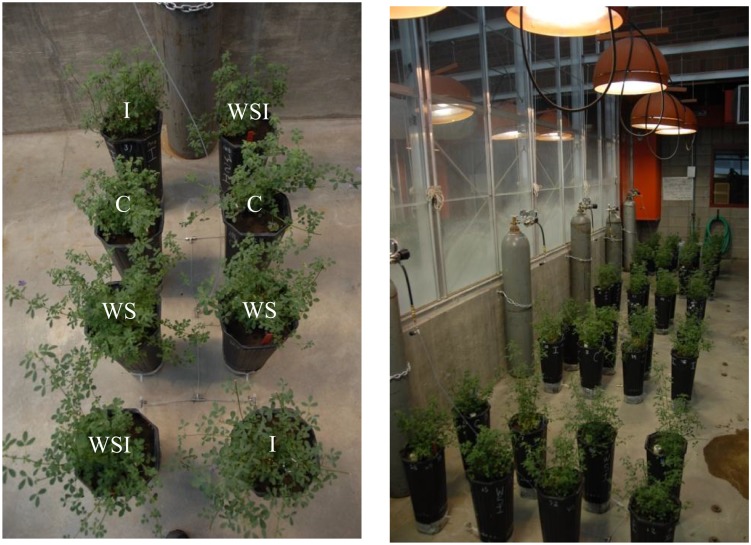
Treatment block design. Single treatment block shown with plumbing and labeled treatments shown in white for each plant (left). Four of five treatment blocks are shown within the greenhouse (right).

Alfalfa plants were randomly assigned to treatment blocks and to discrete positions within each block using a random number generator. Treatments within the blocks were randomly assigned using a stratified randomization developed to maintain equal lengths tubing between the injected plants (Figure 2). The plants that received water continued on the same watering regiment of 300 ^ml^/_day_, while the water-stressed plants were not given any water in order to mimic drought conditions once CO_2_ injection began.

**Figure 3:**
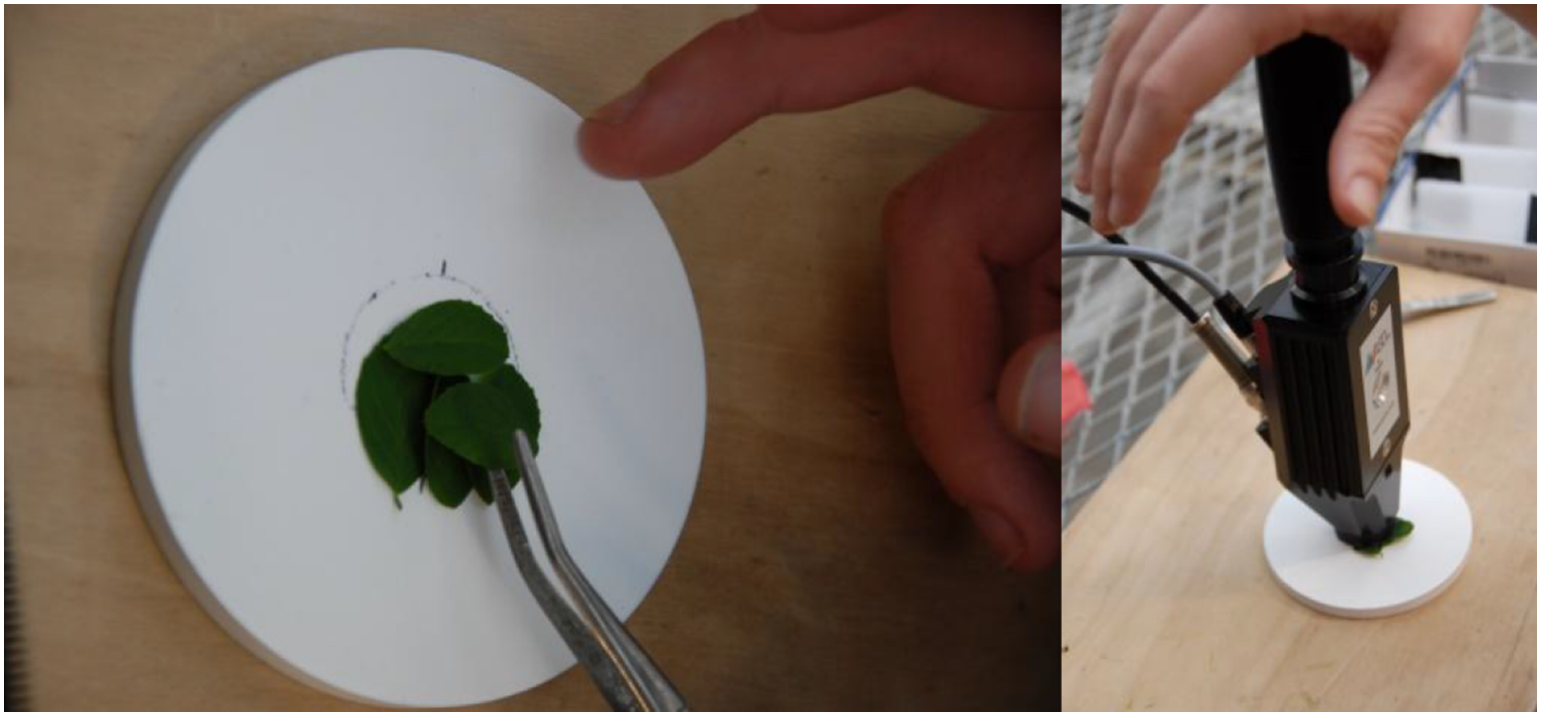
Sampling method. Two dissected alfalfa leaves on the spectralon target (left). The ASD fiber optic and plant probe assembly (right).

Each alfalfa plant was scanned three times a week for three weeks, and all 40 alfalfa plants were sampled twice to obtain two spectral measurements per plant on each scanning date using an ASD Field Spec Pro 350. The ASD is a 16-bit spectrometer that has a spectral range of 350–2500 nm. The spectral resolution is 3 nm at 700 nm, 8.5 nm at 1400 nm, and 6.5 nm at 2100 nm. The sampling interval for the instrument is 1.4 nm at 350–1000 nm and 2 nm at 1000–2500 nm. Prior to interpolation there are a total of 1512 channels that are used in scan acquisition. The instrument takes a scan every 100 ms (ASD Inc.). A single sample consisted of two clipped alfalfa leaves, each containing three leaflets, from a randomly selected stem, determined by the roll of a 4-sided die. Each selected stem was clipped off at the bottom of the sixth node and spectral scans were taken from the four largest leaves on the cut stem. This sampling design intended to measure leaves from both the lower and upper canopy of each alfalfa plant to ensure that stress was not exhibited in one more than the other. The first sample consisted of the upper two biggest leaves and the second was taken from the lower two largest leaves of each cut stem. The leaves were dissected, meaning each leaflet was clipped from each sampled leaf (the leaves were left entire for ease and maximum coverage of the viewing spot later in the experiment, when samples had leaves that were too desiccated and brittle to dissect). The three leaflets from the bottom leaf of the top sample were placed in parallel on a spectralon target overlapped by the three leaflets of the upper leaf from the same sample to achieve total leaf surface coverage of the ASD fiber optic sensor viewing spot, the region from which spectral information is collected. The process was repeated for the lower set of leaves from the same plant for the second sample. These protocols were implemented both to preserve the spatial relationship among the leaf samples, as well as to keep the orientation of the leaves relative to the plant consistent to best simulate what a remote sensing instrument from an airborne platform would view (Figure 3).

The fiber optic cable of the ASD was equipped with a plant probe accessory for leaf-level spectral measurements. The plant probe was 10 in. long and contained an internal, low-intensity halogen bulb, which produces little heat, for collecting spectral scans from vegetation. The viewing geometry of the mounted fiber optic cable created a 10 mm by 13 mm oval viewing spot from which spectral information was collected.

Initial spectral readings were taken for all 40 plants at 1000 hours on February 7, 2011, as a baseline before treatments were applied. CO_2_ injection began at 1400 hours on February 7, 2011. A Vaisala soil probe was used to monitor CO_2_ concentrations over time and to confirm successful injection into the individual plant pots. A Swagelok G2 model variable area flowmeter also was used to measure CO_2_ flux at the quick-connect union for each plant. Injection rates ranged from 0.25 to >5 l/hr for all injected plants. All 40 plants were spectrally scanned on Monday, Wednesday, and Friday for three weeks. Each plant was sampled and scanned on nine dates, except one plant, which was only scanned on eight dates because it was discovered on February 9 that a plant was not receiving CO_2_ injection for some unknown reason. The plant therefore, was swapped out. A total of 18 scans were acquired for all plants, except for the exchanged plant, which had 16 scans. The injection was terminated on February 25, 2011, upon the completion of scanning all the plants earlier that day.

All spectral measurements were in reflectance and were a derived average of 25 individual scans. The instrument was calibrated to set gains and off-sets by an optimization process in the RS^3^ software (ASD Inc.). A dark current was collected subsequently and the sensor was white referenced using a spectralon target. The ASD instrument was periodically recalibrated on each scanning date to ensure accurate and repeatable spectral measurements. A new white reference and dark current reading was made after the acquisition of both spectral scans for a single plant. The ASD was re-optimized upon the completion of acquiring data for an entire treatment block–eight plants.

### Data Analysis

We developed a classification tree model for each individual scanning date to determine the timing and extent to which the different treatment groups were spectrally distinguishable. Classification tree analysis is a non-parametric statistical modeling method that has been successfully used to discriminate vegetation stress classes using hyperspectral data because it can utilize different band combinations to distinguish each class [37]. Classification trees use recursive, decision-based rules that can be interpreted by an analyst. This is important when trying to ascertain how and where the different plant physiological stressors are spectrally distinguished.

Although the ASD contained spectral data within the ultraviolet portion (350–400 nm) of the EM spectrum, these data were removed from further analysis because they were noisy and airborne hyperspectral sensors generally do not collect data at these wavelengths. The ASD measurements for the 400–2500 nm spectral range were output in an ascii format and exported into an Excel spreadsheet. TIBCO Spotfire S+ statistical software package was used to fit classification tree models to each scanning date’s data using treatment group as the response variable and the ASD bands as explanatory (predictor) variables. Additionally, a factor variable indicating the relative position (upper or lower) of each leaf sample was included as a possible predictor variable in case sample location had an influence on treatment response. Cross-validation trees within S+ were used to prune the classification tree models for each date, so that they were unbiased and not over fit [60]. A standard 10-fold cross-validation was performed to determine the appropriate number of terminal nodes to lower deviance among samples, except that, given the small sample size, a script was written in S+ to take a random sample of scans stratified on treatment type (I, WS, WSI, or C) for each cross-validation tree to ensure that a balanced sample for each treatment group was withheld for validation purposes. This process was repeated five times, taking a different stratified random sample for each successive cross-validation tree and the plurality determined the appropriate size for pruning each classification tree. The spectral locations (wavelengths) used to distinguish between treatment groups for each of the binary nodes within each pruned classification tree model were examined to elucidate the spectral regions that best discerned between water stress and stress caused by elevated soil CO_2_ in plants.

A random forest classification [61] also was performed on the individual scanning dates to evaluate the levels of prediction accuracy that could be achieved in discerning water and CO_2_ stress agents. The algorithm is a “bagging” method that takes a bootstrap sample from the data observations to develop a classification tree by using a random subset of all possible explanatory variables (spectral bands) at each binary split [61]. This is done iteratively to form hundreds of classification trees (the forest) and then each observation is classified on the resultant plurality vote of the forest. Random forest models were derived with an ensemble of 500 classification trees with 45 randomly selected explanatory variables (of the possible 2100 spectral bands or the categorical variable indicating relative leaf position in the canopy) to be tried at each binary split within each tree. Variable importance plots from the random forest models were used to highlight the spectral regions used in distinguishing between the different treatment classes. Hyperspectral data have been modeled using random forest to greatly increase classification accuracies, as compared to other methods, as well as provide an unbiased, reliable internal estimate of accuracy for mapping land cover [62] and invasive plants [63]. A disadvantage of random forest is the inability to ascertain the relevance of individual explanatory variables and make meaningful interpretations of the model. Since hundreds of individual trees contribute to a random forest model, the classification results are essentially determined inside a “black box” that is effective for the purpose of prediction, but not for interpreting the decision-based rules that determine those predictions [64].

The lack of replicates in this study forced us to rely on internal measurements of predictive accuracy to evaluate spectral distinguishability. No data were withheld in the construction of the individual classification trees and the random forest classifier withheld approximately one third of each date’s data as a bootstrap (out-of-bag) sample for internal validation within each of the 500 constructed trees. The random forest classifier has proved itself as a robust classification tool that contains an unbiased internal accuracy assessment that does not require a separately withheld dataset for validation [63]. Overall classification accuracies, Kappa statistics (a more robust estimate of classification accuracy because it takes into account chance agreement by differencing the observed accuracy from that of a total random assignment), and individual user’s (mapping errors of commission) and producer’s accuracies (mapping errors of omission) were used to assess predictive capabilities for the class distinctions through time [65].

## Results

**Figure 4:**
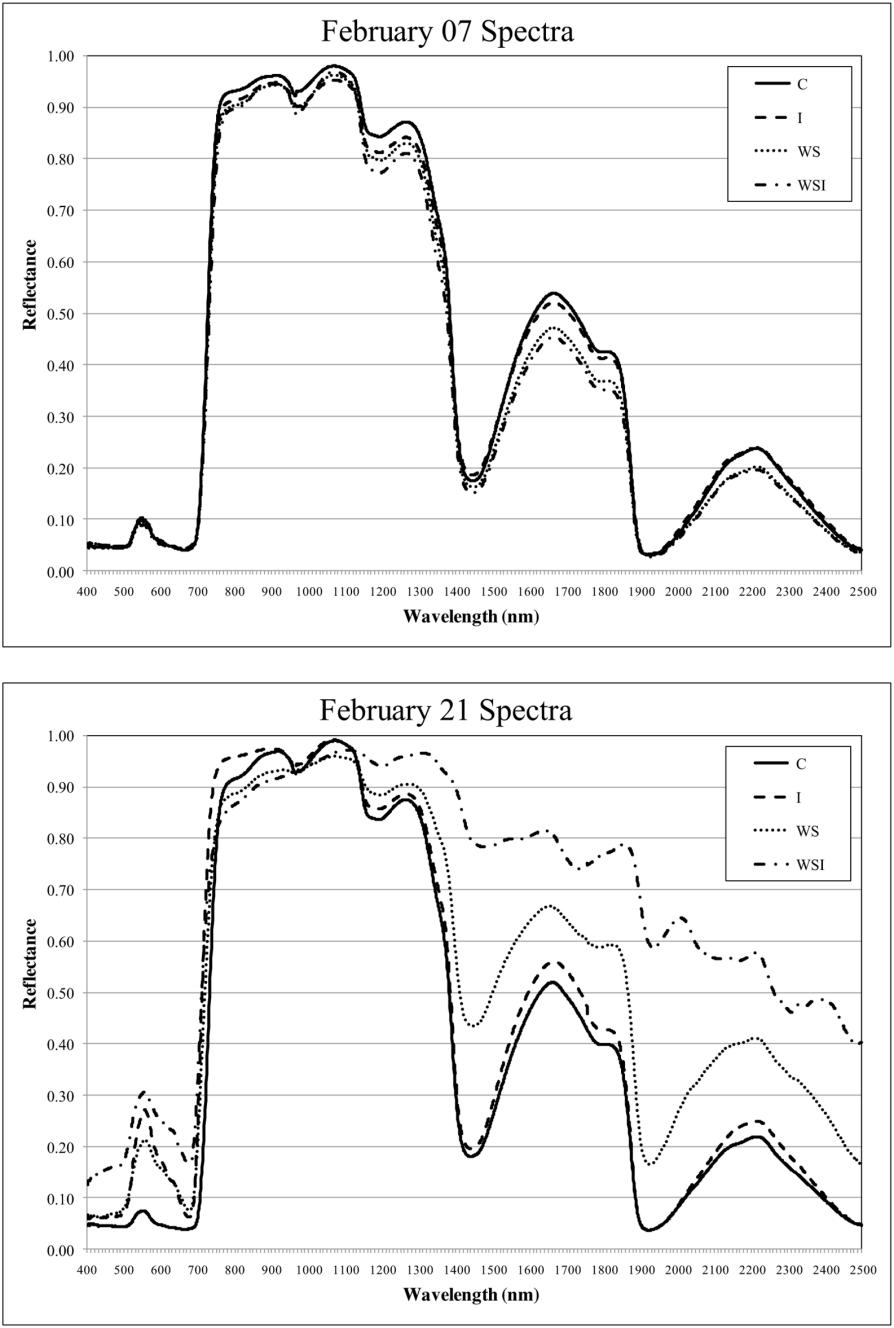
Sample reference spectra for each of the four treatment groups. Spectral signatures for February 07 (before treatment application) and February 21 (when spectral distinction was greatest between treatment groups).

Examination of the individual sample spectral signatures for the different treatment groups illustrated the spectral regions that were critical in their distinction and accurate classification (Figure 4). Sample spectra for each of the treatment groups appeared to exhibit similar reflectance properties in the visible wavelengths, while subtle spectral deviations occurred in the near and SWIR peak reflectance features before treatments were applied, on February 07. The stressed sample spectra, in contrast, appeared to exhibit increased reflectance in the visible spectrum, especially within the visible green, as compared to the C class on February 21, at the height of spectral distinction among treatment groups. Water stressed samples exhibited increased reflectance in the SWIR as compared to the C and I classes. The WSI class predominantly exhibited higher reflectance in the visible and SWIR regions compared to all other samples. The red edge spectral boundary shifted towards shorter wavelengths (the “blue shift”) for stressed samples after four days of treatment application, however, visual symptoms of stress were also observed on this day. The blue shift was not uniquely expressed among stressed treatment groups.

### Classification Tree Analysis

**Figure 5:**
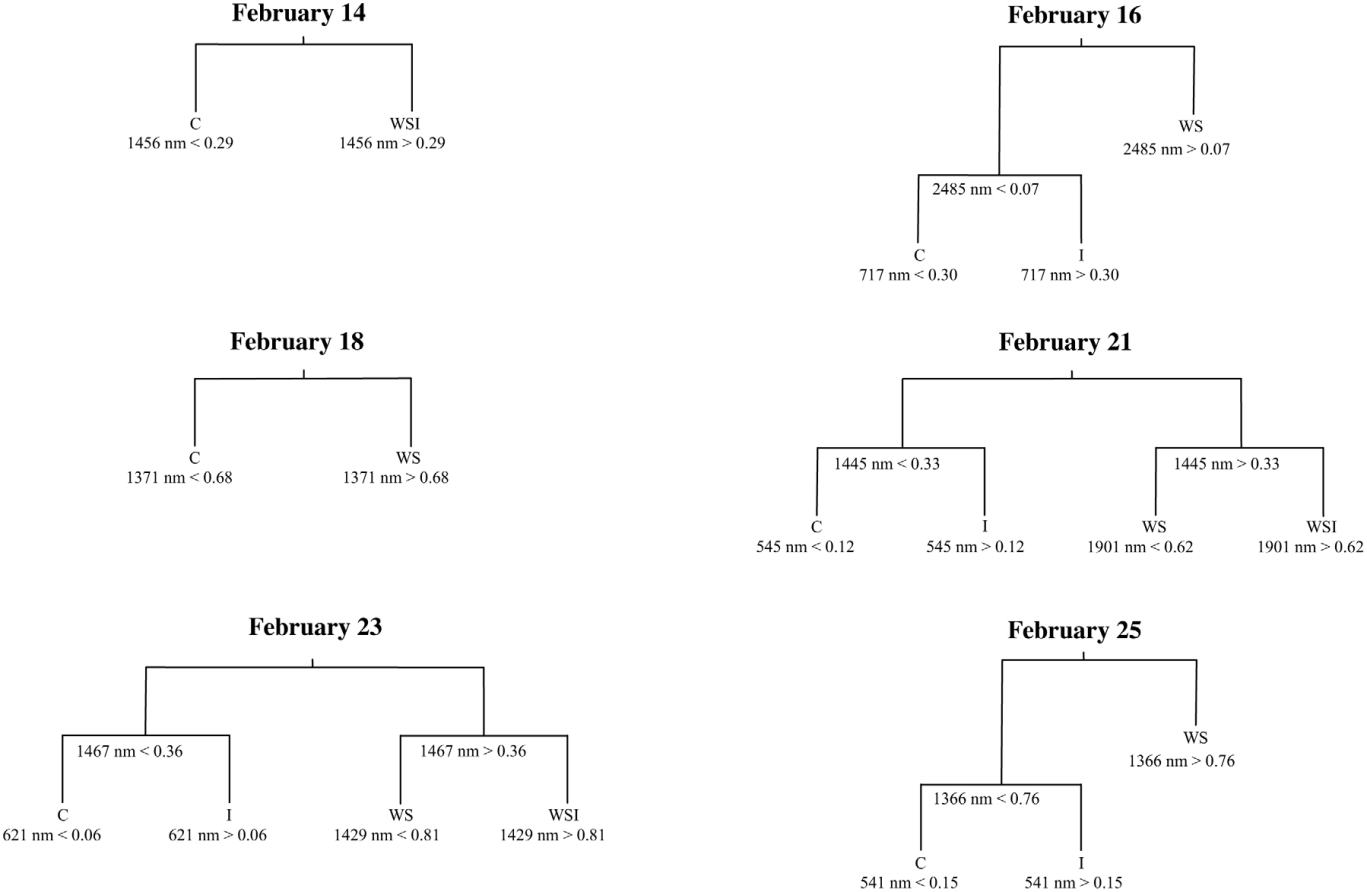
Pruned single date classification trees. Each tree shows the utilized spectral bands (by central band wavelength) and reflectance levels for each splitting rule. Splitting rules apply to the left branches of the tree. C = control class; I = CO_2_ injection class; WS = water stress class; and WSI = water stress and CO_2_ injection interaction class.

Cross-validation results determined that four terminal nodes were appropriate for February 21 and 23, otherwise the classification trees were pruned smaller to ensure they were not over fit (Table 1). Only the February 21 and 23 classification tree models, therefore, distinguished among all four treatment groups. A decision split or node was not justified for the classification tree models during the first week of the experiment. Not until February 14 were two or more terminal nodes warranted (Figure 5). The categorical variable indicating relative leaf sample position within each alfalfa canopy was never used as a splitting rule for any of the classification trees.

**Table 1:** Number of terminal nodes determined by the cross-validation results for the single date classification tree models.

Cross-Validation Tree Results						
Date	x1	x2	x3	x4	x5	Plurality
2/7	1	1	1	1	1	1
2/9	1	1	1	1	1	1
2/11	1	1	2	2	1	1
2/14	2	1	2	2	1	2
2/16	3	3	3	3	2	3
2/18	2	2	5	2	2	2
2/21	4	4	4	4	4	4
2/23	4	4	4	4	4	4
2/25	3	2	2	3	3	3

The plurality of the five cross-validation trials determined the appropriate size of the final trees.

WS and WSI classes exhibited greater reflectance than the C and I classes within the water absorption bands near the 1400, 1900, and 2500 nm wavelengths. These distinctions in the SWIR infrared regions began on February 14 and persisted until the end of the experiment. Second level distinctions (decisions in trees with three or more terminal nodes) were made within the red edge at the 717 nm wavelength, where greater reflectance was exhibited by leaf samples from the I class as compared to the C class. The I class also exhibited greater reflectance in the visible green-visible orange at the 545, 621, and 541 nm wavelengths as compared to the C class. The WSI interaction group exhibited compound stress effects with greater reflectance as compared to the WS class in the water absorption bands at 1429 nm and 1901 nm.

Confusion early in the experiment, from February 14–18, occurred almost exclusively between (1) I and C classes, and (2) WSI and WS classes (Table 2). The C class was accurately distinguished by the second week of stress treatment application (user’s and producer’s accuracies ≥91%). Spectral differentiation between treatment groups was greatest on February 21 and February 23 (after two weeks of treatment application) with overall classification accuracies of 90% (Kappa of 0.87) and 81% (Kappa 0.75), respectively. Confusion during maximum spectral distinction primarily occurred (1) with I samples being classified as WS, and (2) WS being classified as WSI. Producer’s accuracies for the I class were 80% and 55%, while the WSI class had perfect producer’s accuracies (100%) on both February 21 and 23. Overall classification accuracy dropped to 66% (Kappa of 0.55) on the last scanning date, February 25.

**Table 2:** Internal classification accuracies and Kappa statistics for each single date classification tree model containing at least two terminal nodes.

February 14, 2011	C	I	WS	WSI	User’s Accuracy	February 16, 2011	C	I	WS	WSI	User’s Accuracy
C	20	20	6	3	41%	C	16	0	0	0	100%
I	0	0	0	0	na	I	4	19	0	0	83%
WS	0	0	0	0	na	WS	0	1	20	20	49%
WSI	0	0	14	17	55%	WSI	0	0	0	0	na
Producer’s Accuracy	100%	0%	0%	85%	Overall Accuracy 46%	Producer’s Accuracy	80%	95%	100%	0%	Overall Accuracy 69%
Kappa Statistic	0.28					Kappa Statistic	0.58				
**February 18, 2011**	**C**	**I**	**WS**	**WSI**	**User’s Accuracy**	**February 21, 2011**	**C**	**I**	**WS**	**WSI**	**User’s Accuracy**
C	20	20	0	0	50%	C	20	0	0	0	100%
I	0	0	0	0	na	I	0	16	0	0	100%
WS	0	0	20	20	50%	WS	0	4	16	0	80%
WSI	0	0	0	0	na	WSI	0	0	4	20	83%
Producer’s Accuracy	100%	0%	100%	0%	Overall Accuracy 50%	Producer’s Accuracy	100%	80%	80%	100%	Overall Accuracy 90%
Kappa Statistic	0.33					Kappa Statistic	0.87				
**February 23, 2011**	**C**	**I**	**WS**	**WSI**	**User’s Accuracy**	**February 25, 2011**	**C**	**I**	**WS**	**WSI**	**User’s Accuracy**
C	20	2	0	0	91%	C	20	1	0	0	95%
I	0	11	0	0	100%	I	0	13	0	0	100%
WS	0	6	14	0	70%	WS	0	6	20	20	43%
WSI	0	1	6	20	74%	WSI	0	0	0	0	na
Producer’s Accuracy	100%	55%	70%	100%	Overall Accuracy 81%	Producer’s Accuracy	100%	65%	100%	0%	Overall Accuracy 66%
Kappa Statistic	0.75					Kappa Statistic	0.55				

Treatment group columns represent the reference data while the rows represent the classified data.

### Random Forest Analysis

**Figure 6:**
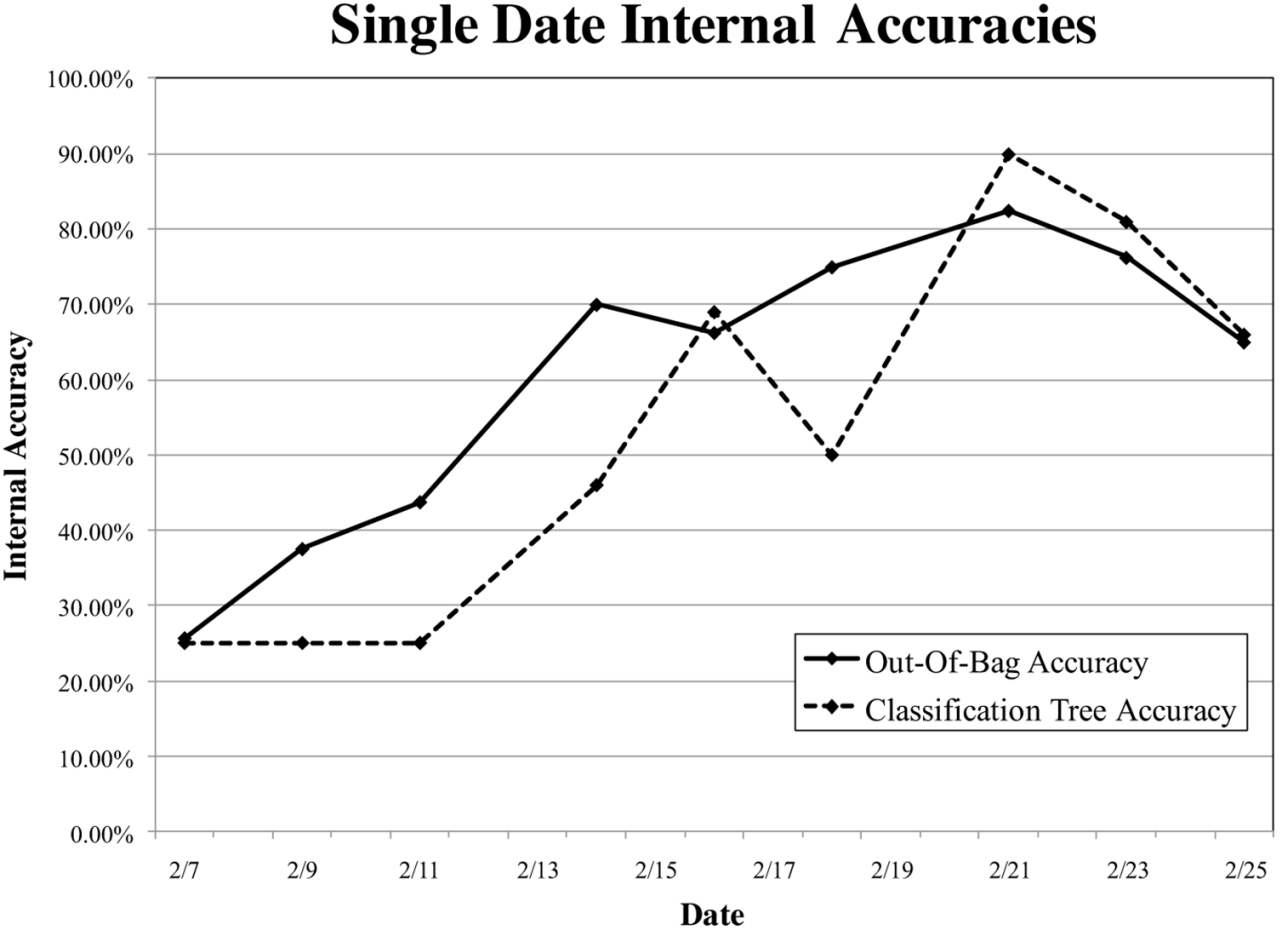
Overall internal accuracies for each random forest and classification tree model over the course of the experiment. Note that the February 7–11 classification trees did not contain ≥ two terminal nodes.

The trend in accuracy of the random forest models throughout the time series was similar to that of the classification tree models (Figure 6). Out-of-bag accuracy predominantly exhibited a curvilinear trend with increasing out-of-bag accuracy from 25% (Kappa of 0) on February 7 (before treatments were applied) to peak classification accuracy of 83%, (Kappa of 0.77) on February 21. Subsequently, classification accuracy decreased. By February 25 out-of-bag accuracy had dropped to 65% (Kappa of 0.53). The C treatment group was best distinguished with user’s and producer’s accuracy ≥80% after one week of treatment application (Table 3). Most class confusion existed between WSI and WS treatment groups, once overall out-of-bag accuracy was ≥65% (February 14–25). I class producer’s accuracy was ≥65% from February 14 onward. WSI class producer’s accuracy was 90% for February 21 and 23, but was much lower on other dates.

**Table 3:** Out-of-bag accuracies and Kappa statistics for each single date random forest model.

February 7, 2011	C	I	WS	WSI	User’s Accuracy	February 9, 2011	C	I	WS	WSI	User’s Accuracy
C	5	2	3	5	33%	C	9	2	4	2	53%
I	3	4	6	2	27%	I	4	9	6	8	33%
WS	4	6	5	7	23%	WS	3	4	7	5	37%
WSI	8	6	6	6	23%	WSI	4	5	3	5	29%
Producer’s Accuracy	25%	22%	25%	30%	Overall Accuracy 26%	Producer’s Accuracy	45%	45%	35%	25%	Overall Accuracy 38%
Kappa Statistic	0					Kappa Statistic	0.17				
**February 11, 2011**	**C**	**I**	**WS**	**WSI**	**User’s Accuracy**	**February 14, 2011**	**C**	**I**	**WS**	**WSI**	**User’s Accuracy**
C	9	10	2	3	38%	C	17	2	2	0	81%
I	10	7	3	3	30%	I	2	13	1	2	72%
WS	0	2	10	5	59%	WS	1	2	12	4	63%
WSI	1	1	5	9	56%	WSI	0	3	5	14	64%
Producer**’**s Accuracy	45%	35%	50%	45%	Overall Accuracy 44%	Producer’s Accuracy	85%	65%	60%	70%	Overall Accuracy 70%
Kappa Statistic	0.25					Kappa Statistic	0.6				
**February 16, 2011**	**C**	**I**	**WS**	**WSI**	**User’s Accuracy**	**February 18, 2011**	**C**	**I**	**WS**	**WSI**	**User’s Accuracy**
C	16	4	0	0	80%	C	17	4	0	0	81%
I	4	15	1	1	71%	I	3	14	1	0	78%
WS	0	1	13	10	54%	WS	0	2	14	5	67%
WSI	0	0	6	9	60%	WSI	0	0	5	15	75%
Producer’s Accuracy	80%	75%	65%	45%	Overall Accuracy 66%	Producer’s Accuracy	85%	70%	70%	75%	Overall Accuracy 75%
Kappa Statistic	0.55					Kappa Statistic	0.67				
**February 21, 2011**	**C**	**I**	**WS**	**WSI**	**User’s Accuracy**	**February 23, 2011**	**C**	**I**	**WS**	**WSI**	**User’s Accuracy**
C	19	1	0	0	95%	C	19	2	0	0	90%
I	1	16	2	0	84%	I	1	14	3	0	78%
WS	0	3	13	2	72%	WS	0	4	10	2	63%
WSI	0	0	5	18	78%	WSI	0	0	7	18	72%
Producer’s Accuracy	95%	80%	65%	90%	Overall Accuracy 83%	Producer’s Accuracy	95%	70%	50%	90%	Overall Accuracy 76%
Kappa Statistic	0.77					Kappa Statistic	0.68				
**February 25, 2011**	**C**	**I**	**WS**	**WSI**	**User’s Accuracy**						
C	19	2	1	0	86%						
I	1	15	2	0	83%						
WS	0	1	8	10	42%						
WSI	0	2	9	10	48%						
Producer’s Accuracy	95%	75%	40%	50%	Overall Accuracy 65%						
Kappa Statistic	0.53										

Treatment group columns represent the reference data while the rows represent the classified data.

**Figure 7:**
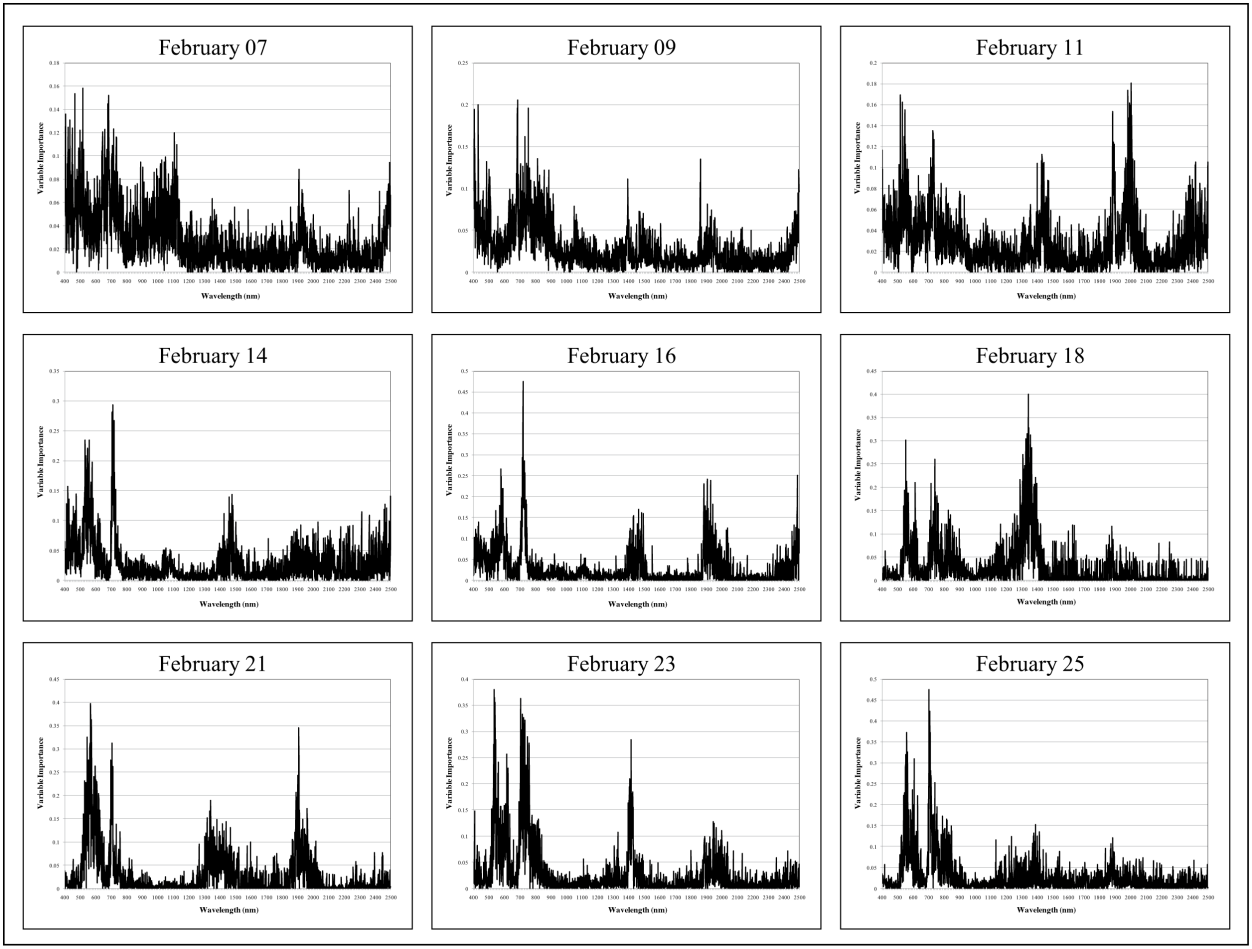
Variable importance plots for the single date random forest models. Note that the leaf position categorical variable is not included.

The variable importance plots provided a visual display of the spectral regions that were the most influential in single date random forest model prediction (Figure 7). The variable importance plots were very noisy before there was spectral discernability among treatment groups. Spectral regions became more clearly defined as classification accuracies increased. The red edge region (700–750 nm) was shown to be the most important on February 14 and 16. On February 18, variable importance was predominantly placed on the spectral wavelengths centered on approximately 1400 nm. During maximum spectral distinction among treatment groups, variable importance was placed (1) in the visible green-yellow portion of the EM spectrum centered at approximately 550 nm; (2) the red edge (700–750 nm); and (3) the three water absorption features centered at approximately 1400, 1900, and 2500 nm. On February 25, the last scanning date, more emphasis was placed on the visible and NIR spectral regions rather than the water absorption features. The categorical variable indicating relative leaf sample position within each alfalfa canopy was never deemed important in any of the random forest classification models (variable importance<0.01).

## Discussion

Our analyses demonstrated that hyperspectral spectrometry could distinguish between CO_2_ stressed and healthy alfalfa leaves (I v. C). Furthermore, the spectral distinction of plant stress caused by elevated soil CO_2_, water stress conditions, and their interaction was possible at certain times during the greenhouse experiment. Those findings have significant implications for the use of hyperspectral imaging to monitor GCS sites for CO_2_ leaks given that water stress is a prevalent environmental condition and could be a confounding factor. Remote sensing monitoring of CO_2_ leaks amid a landscape of patchy, water stressed vegetation would most likely require differentiation of CO_2_ and water stress (I v. WS), whereas during widespread drought, vegetation stressed by both CO_2_ and water stress would need to be spectrally distinct from surrounding vegetation (WSI v. WS).

Confusion predominantly occurred between (1) I and C classes, (2) I and WS classes, and (3) WSI and WS classes, which could be problematic for CO_2_ leak detection in a GCS monitoring context. Mapping errors of omission (low producer’s accuracy) for CO_2_ stressed vegetation (I and WSI) would be less acceptable from a hyperspectral monitoring standpoint, while mapping errors of commission (low user’s accuracy) would be more tolerable, since a CO_2_ leak would be less likely to go unidentified. High commission error and low omission error would result in the overestimation of CO_2_ leaks, perhaps wasting labor resources for ground verification, however, CO_2_ leaks would less likely be missed.

### CO_2_ Leak Detection When Water Is Not Limiting

Hyperspectral monitoring of GCS sites when water stressed vegetation is not present would consider the spectral discrimination between I and C classes. Spectral distinctions in the red edge, visible green, and visible orange regions were used to distinguish I from C classes. Rules discerned increased green reflectance within the I class multiple times near 550 nm, a spectral region sensitive to xanthophyll pigment activities associated with photosynthetic efficiency [42], [59] and changes to chlorophyll content [46]. Additionally, increased reflectance at the 620 nm and 717 nm wavelengths distinguished I from C classes. These are also spectral regions that are known to be highly sensitive to chlorophyll *a* content and absorption [32]. The primary spectral response of the I class to elevated soil CO_2_ was increased reflectance within the visible green–orange spectrum.

The I class was distinguished from all treatment groups with user’s and producer’s accuracies ≥70% beginning February 16, after ten days of CO_2_ injection. Classification accuracies of the I class remained high until the end of the experiment, suggesting that a detectable stress signal caused by a CO_2_ leak could persist for a substantial period of time. I and C classes were likely confounded in the experiment because some alfalfa plants received comparatively higher CO_2_ injection rates due to minor differences in gas flow dynamics influenced by plant pot plumbing geometry and soil compaction. Severity and timing of stress caused by CO_2_ injection therefore, varied. Healthy plants also appeared to be disproportionately targeted by a two-spotted spider mite infestation, which possibly caused a stress response that was most spectrally similar to stress caused by elevated soil CO_2_ given that the insects caused a discoloration in the leaves most similar to the chlorosis observed in the alfalfa plants receiving CO_2_ injection. The two-spotted spider mites were treated with numerous pesticides and biological controls, however, they probably remained an influential stress factor for healthier alfalfa plants. The results of the random forest classification demonstrated that stressed and non-stressed vegetation were accurately distinguished after one week of treatment application (C class accuracies ≥80%). Spectral differentiation among the stress classes was more difficult and time dependent, however.

### CO_2_ Leak Detection When Soil Moisture Is Spatially Variable

Identification of a CO_2_ leak would likely require water stress to be spectrally distinguished from stress caused by elevated soil CO_2_ because soil moisture availability is highly variable across most landscapes [66]–[67]. Increased reflectance within the water absorption regions near 1400 and 2500 nm was the most important characteristic that distinguished water stressed classes (WSI and WS) from C and I classes. The primary spectral response of leaf dehydration occurs within the water absorption regions (near 1400, 1900, and 2500 nm) that are sensitive to leaf water content [53].

Patterns of variable importance for each of the random forest models identified discrete spectral regions that were particularly useful in discriminating among treatment groups. The red edge and the visible green-visible orange spectral regions were at least as important as the water absorption bands for distinguishing among the different classes. However, the “black box” nature of the random forest classifier did not allow for specific interpretation of the spectral distinctions made by the model [64]. It is possible that the variable importance within the visible and NIR wavelengths was related to distinguishing between the I and C classes given that these two treatment groups appeared to be spectrally similar in the SWIR region, while the water stress classes were likely separated by distinctions in the water absorption regions. Eventually increased reflectance in the visible, NIR, and SWIR regions can be exhibited by all stressed plants regardless of the cause, however [53].

Alfalfa plants of the I treatment group exhibited visual stress predominantly as chlorosis (yellowing of plant leaves due to lack of chlorophyll production), although some CO_2_ injected plants’ leaves did become desiccated by the end of the experiment, suggesting leaf water loss. Leaves of the WS treatment group generally exhibited a lack of vigor (leaves were droopy) early in the experiment, and eventually, they became dessicated and brittle as the water stress severity increased. Overall these classes were distinguished reasonably well. Only minor confusion occurred probably because of similar reflectance properties in the visible wavelengths due to eventual chlorophyll loss to water stressed samples and due to eventual leaf moisture loss, expressed as increased reflectance in the water absorption bands of I class samples [53].

### CO_2_ Leak Detection During Widespread Drought

Remote monitoring of GCS sites could require CO_2_ leaks to be spectrally distinguished during drought conditions when all vegetation is subjected to water stress. Elevated soil CO_2_ would thereby have to interact with water stress to cause a compound stress response in vegetation that was spectrally discernable. Greater reflectance in the water absorption bands near 1400 nm was used to distinguish WSI from WS classes, perhaps in response to diminished water-use-efficiency caused by comparatively faster leaf moisture loss or reduced water uptake at the root level. This was consistent with a compound stress interaction being exhibited by the WSI class. Treatment class accuracies indicated that alfalfa plants that went without water for one week exhibited water stress that became spectrally discernable on February 14. The confusion existed primarily *between* them and not with the other classes. Individual WS and WSI class accuracies, therefore, were low. This was consistent with a water stress signal that was more easily discerned because of the discrete water absorption features where leaf reflectance is directly related to leaf moisture content. Spectral distinction of the WSI class remained relatively convoluted with the WS class throughout the experiment. The WSI class was distinguished with high producer’s accuracies (low omission error) on February 21 and 23, perhaps suggesting that vegetation could exhibit stress in response to a CO_2_ leak that could be distinguished during drought conditions, albeit a narrow time window.

The plant leaves of the water stressed treatment groups became desiccated and brittle as the experiment progressed. These plants eventually began to die. Spectral distinction within the water absorption bands was probably difficult due to the extreme leaf moisture loss caused by severe drought by the end of the experiment. Little variable importance was placed on the water absorption features in the last classification date (February 25), where the water stressed treatment groups were poorly distinguished (accuracies≤50%), while the I and C treatment groups were classified with reasonable accuracy (accuracies ≥75%). This was evidence that the alfalfa plants’ response to severe drought surpassed a stress threshold that rendered the CO_2_ stress signal spectrally indistinguishable.

### Pre-visual Plant Stress Detection

Pre-visual stress detection would be a critical attribute and an advantage to using hyperspectral remote sensing for early CO_2_ leak detection at a GCS site. February 11 was the first day that subtle visual evidence of stress to some of the WSI and I treatment group plants was evident. Early visual symptoms of stress (i.e., chlorosis and languid leaves) were confined to older leaves located lower on the stems within the alfalfa plant canopies. Visible stress was most prominent within the WSI treatment group, which exhibited the compound effects of both CO_2_ injection and lack of water. The accurate classification of the WSI samples did not occur until February 14, one week into the experiment, even though stress was noted visually three days before that time. The blue shift of the red edge towards shorter wavelengths has been associated with the pre-visual detection of plant stress [32], [55], [57], and was observed for some sample spectra by February 11. Blue shift spectral characteristics were similar in depth and slope for all of the stressed classes and, therefore, could not be used to distinguish among stress agents. This experiment provided no evidence that the pre-visual detection of plant stress was possible with hyperspectral data.

### Summary

The primary conclusions that can be drawn from this greenhouse experiment are (1) that plant stress caused by elevated soil CO_2_ was spectrally detected, probably in the visible spectrum, after approximately one week of CO_2_ injection through the end of the experiment (random forest I class accuracies ≥65%). This is potentially, evidence that a spectral stress signal caused by elevated soil CO_2_ could persist for a substantial period of time; (2) CO_2_ and water stress were spectrally distinguishable; and (3) Elevated soil CO_2_ appeared to cause a compound stress response detected in plants that were already water stressed, however, there was a relatively narrow time window when the WSI class was spectrally distinguished from the WS class. This was indicative of a time-dependent compound stress response caused by the interaction of elevated soil CO_2_ and drought.

Although the results were variable between the individual classification tree and random forest classifier results, they both illustrated the same pattern of predictive success for plant stress detection. Differences between the two modeling strategies were likely due to the small sample size relative to the high dimensionality of the hyperspectral data. There were a total of 40 plants, 10 plants per class, as compared to the 1512 possible predictor variables. Depending on which samples were withheld for validation purposes during model development, variability did occur between outputs. Additionally, the random forest classifier uses a randomized node optimization which would introduce additional variability. Therefore, these differences were expected especially when using two distinctly different classification methods.

These results suggest that remote sensing may be used to monitor GCS sites for CO_2_ leaks. Detection of a CO_2_ leak when the availability of soil water is highly variable across space might be possible even if there are co-occurring patches of water stressed vegetation. Plants appear to hit a stress threshold, however, that renders spectral detection of a CO_2_ leak unlikely during severe drought conditions. Regardless, this research demonstrated the necessity for remote sensing instruments to be spectrally sensitive to SWIR reflectance in order to accurately distinguish CO_2_ and water stress.

Even though pre-visual stress detection was not possible at the leaf-scale, the early detection of stress caused by elevated soil CO_2_ was achieved. Aerial hyperspectral imaging has been demonstrated to be effective for early stress detection at broader spatial scales [37] and the random forest classifier has been used to successfully map invasive plants in a natural setting [63]. Monitoring for CO_2_ leaks at GCS sites will possibly require large area coverage, which will only be possible from an airborne platform. Hyperspectral instruments with high spatial resolution optics for canopy-level monitoring at altitude, therefore, might be important for the early detection of CO_2_ leaks. The high dimensional nature of hyperspectral data will require robust data analysis methods that can detect a plant stress signature or signal within large datasets. Different data mining tools exist that could both reduce the dimensionality of hyperspectral data and discern the targeted CO_2_ stress signature in an aerial monitoring context. For example, an orthogonal operator has been used to reduce the dimensionality of hyperspectral data while retaining the meaningful signature of interest [68]–[69]. Additionally, a hypergraph method that jointly relates the spatial context of image pixels to their associated spectral characteristics has been demonstrated to be effective in hyperspectral image classification and may be useful in detecting pockets of plant stress caused by elevated soil CO_2_ [70]. Further exploration of different data analysis methods will be essential to optimize a workflow that would enable an analyst to efficiently assimilate large datasets and effectively detect a CO_2_ leak in a GCS monitoring context.

Additional research is warranted to understand the extent to which elevated soil CO_2_ stress could be detected at the canopy-level using airborne remote sensing, especially when water stress conditions exist. Spectral differentiation between CO_2_ and water stressed vegetation would likely include spectral distinctions within the water absorption features. Spectral data acquired within the water absorption regions from an airborne remote sensing platform would likely be attenuated by atmospheric water vapor, perhaps further confounding CO_2_ leak detection [47], [54]. Snow cover and drought are going to limit the applicability of remote sensing data in temperate climates, therefore hyperspectral monitoring will need to be used in conjunction with other monitoring methods to obtain adequate temporal coverage of GCS sites. Improved understanding of vegetation spectral responses to stress caused by plant senescence will also be critical in determining the seasonal timing in which remote sensing data are appropriate for the detection of plant stress caused by an underground CO_2_ leak.
